# Taurine and Polyphenol Complex Repaired Epidermal Keratinocyte Wounds by Regulating IL8 and TIMP2 Expression

**DOI:** 10.3390/cimb46080512

**Published:** 2024-08-08

**Authors:** Sooyeon Lee, Jae Young Shin, Oh Sun Kwon, Seung-Hyun Jun, Nae-Gyu Kang

**Affiliations:** LG Household & Health Care (LG H&H) R&D Center, 70, Magokjoongang 10-ro, Gangseo-gu, Seoul 07795, Republic of Korea; sooylee@lghnh.com (S.L.); sjy2811@lghnh.com (J.Y.S.); kos0119@lghnh.com (O.S.K.); junsh@lghnh.com (S.-H.J.)

**Keywords:** chlorogenic acid, taurine, HaCaT, wound healing, acne scar, tight junction

## Abstract

The healing process after acne lesion extraction provides a miniature model to study skin wound repair mechanisms. In this study, we aimed to identify solutions for acne scars that frequently occur on our faces. We performed acne scar cytokine profiling and found that Interleukin 8 (IL8) and Tissue inhibitor of metalloproteinases 2 (TIMP2) were significant factors at the wounded site. The effect of chlorogenic acid and taurine on human epidermal cells and irritated human skin was investigated. Chlorogenic acid and taurine regulated IL8 and TIMP2 expression and accelerated keratinocyte proliferation. Moreover, tight junction protein expression was upregulated by chlorogenic acid and taurine synergistically. Further, these compounds modulated the expression of several inflammatory cytokines (IL1α, IL1β, and IL6) and skin hydration related factor (hyaluronan synthase 3; HAS3). Thus, chlorogenic acid and taurine may exert their effects during the late stages of wound healing rather than the initial phase. In vivo experiments using SLS-induced wounds demonstrated the efficacy of chlorogenic acid and taurine treatment compared to natural healing, reduced erythema, and restored barrier function. Skin ultrasound analysis revealed their potential to promote denser skin recovery. Therefore, the wound-restoring effect of chlorogenic acid and taurine was exerted by suppression of inflammatory cytokines, and induction of cell proliferation, tight junction expression, and remodeling factors.

## 1. Introduction

Among several types of skin wounds, acne scars are the most prevalent. Once skin wounds occur, inflammatory cytokines are secreted from immune cells to protect the injured site from bacterial infections [1]. These initial stages of wound healing are crucial to establish a proper environment for tissue repair. However, an imbalance in repair factors (i.e., inflammatory cytokines, reactive oxygen species (ROS), proteases, and extracellular matrix (ECM) component levels) can result in several skin impairments [2,3]. In particular, the formation of chronic wounds due to excessive cytokine secretion remains an issue, as it is difficult to control the subsequent inflammation in these wounds [4].

A few pathogenic bacteria (i.e., *Staphylococcus aureus* and *Pseudomonas aeruginosa*) can infect injuries [5]. However, inflammatory cytokines secreted from *Cutibacterium acnes* (*C. acnes*) impact the wound-healing process, as *C. acnes* is present in the largest proportion on the skin [6,7]. High concentrations of *C. acnes* at the sites of injury cause chronic inflammation, post inflammatory hyperpigmentation (PIH), and impaired collagen synthesis, delaying wound closure [8,9,10]. Recent proteomics studies revealed that extracellular vesicles (EVs) from *C. acnes* triggered the production of inflammatory factors such as IL6, IL8, TNF-α, and GM-CSF in the skin and reduced sebum production [11,12]. However, the exact *C. acnes* subtypes and cytokines associated with wound closure after popping pimples are still unclear.

Taurine (2-aminoethanesulfonic acid), a type of beta-amino acid, is a promising anti-oxidant that relieves inflammation throughout the body [13]. Specifically, taurine sustains a normal electron transport chain, maintains glutathione stores, upregulates antioxidant responses, increases membrane stability, eliminates inflammation, and prevents calcium accumulation [14,15,16,17,18]. Structurally, due to high hydrophilicity (sulfonic acid), taurine has low permeability to cellular membranes. However, using the sodium/chloride-dependent taurine transporter (TauT), taurine passes through the lipophilic membrane efficiently. For these reasons, taurine is regarded as a regulator of homeostasis in the skin [19,20]. A previous study reported that the content of taurine in the skin diminished gradually due to aging, implicating the necessity of taurine supplementation to maintain skin repair potential [21].

To evaluate ROS scavenging functions which inhibit inflammatory cytokine recruit-ment during the wound-healing process, we identified several polyphenol compounds [22]. Among these polyphenols, chlorogenic acid, which mainly functions as an antioxidant in cases of bacterial infection, in tumors, and during inflammation, was recently reported to be a modulator of taurine metabolism [23]. Taurine Upregulated Gene 1 (*TUG1*) was identified in a genomic screen for genes upregulated in response to taurine treatment and its expression is upregulated by chlorogenic acid which relieves oxidative stress. The role of *TUG1* in the skin is not well characterized, although the downregulation of *TUG1* was shown to induce melanogenesis [24].

In this study, we aimed to identify factors that influence the wound-healing process by analyzing the effects of diverse *C. acnes* strains such as RT1, RT4, RT5, and RT6 on skin cells and evaluate novel compounds that are capable of modulating the expression and activity of multiple factors. Further, we aimed to investigate the impact of chlorogenic acid and taurine treatment such as synergistic wound healing, hydration, anti-inflammation, and tight junction regulation in human epidermal keratinocytes in vitro and in vivo.

## 2. Materials and Methods

### 2.1. HaCaT Cell Culture and Preparation of Irritant

HaCaT cells (Addexbio technologies; San Diego, CA, USA) are a human epidermal keratinocyte cell line. The cells were cultured in Dulbecco’s Modified Eagle Medium (DMEM), which contained 10% fetal bovine serum (FBS, Gibco; Grand Island, NY, USA), 100 U/mL penicillin, and 100 μg/mL streptomycin (Gibco). The cells were cultured in a humidified environment with 5% CO_2_ and 95% air at 37 °C. The cells were harvested after treatment with 0.05% Trypsin-EDTA (Gibco) once the culture reached 80–90% confluency. Cells obtained from passages 3–15 were used in this study.

### 2.2. C. acnes Culture and Preparation of Irritant

*C. acnes* RT4 (HL053PA1), RT5 (HL043PA1), and RT6 (HL110PA3) were cultured in Bacto™ Brain Heart Infusion media (BD Diagnostics; Franklin Lakes, NJ, USA) under anaerobic conditions at 37 °C. As an irritant, the cultures were heat-killed by incubation at 65 °C for 1 h.

### 2.3. Cytokine Array Analysis

HaCaT cells (5 × 10^5^ cells/plate) were seeded on 75T plates and cultured for 72 h. Next, heat-killed *C. acnes* RT 4, 5, 6 diluted in 5 mL of DMEM (1 × 10^7^ CFU/mL) were added to the cells and incubated for 24 h. The supernatant was collected and cytokine expression was analyzed using the Human Inflammation Antibody Array (Ab134003, abcam; Cambridge, UK), which can detect 40 human inflammatory factors simultaneously, as per the manufacturer’s instructions.

The array signal intensity was analyzed using an iBright 1500 imaging system (Invitrogen; Carlsbad, CA, USA). The resulting images were evaluated using the ImageJ/Fiji^®^ (Version: 1.51j8) plugin Protein Array Analyzer (Version: 1.1.c, Gilles Carpentier Research) to quantify the spot densities. After subtracting the background signals, the data were normalized by the values of the untreated control group.

### 2.4. Measurement of IL8 and TIMP2 Expression Levels

To measure the IL8/TIMP2 expression level on the surface of human skin via a non-invasive technique, two healthy individuals, and two patients with acne were recruited. All subjects were Korean, and patients with acne were selected based on an Investigator’s Global Assessment (IGA) grade 2 (mild) to 3 (moderate) and those who were between 25–40 years of age. Skin cytokine sampling was performed from the left cheek of healthy individuals and on mild-to-moderate acne lesions on the face of individuals with acne by rubbing a nylon flocked swab (eSwab kit 480CE, Copan, Murrieta, CA, USA) prehumidified in phosphate-buffered saline (PBS; Gibco; Grand Island, NY, USA). All samples were taken from a cross-sectional area of 1 cm^2^. In patients with acne, samples were collected from a 1 cm^2^ area surrounding an inflammatory lesion (papule) and a post-inflammatory hyperpigmentation lesion. Swab samples were obtained during the same visit by rubbing for one minute. Samples were then individually soaked in PBS with 0.05% Tween^®^ 20 and 0.5 mM EDTA. The samples were subsequently vortexed for 30 min, cooled down on ice for 30 min, sonicated for 1 h, and again vortexed for 30 min and used for ELISA. For analysis of the in vitro expression levels of IL8/TIMP2, HaCaT cells (1 × 10^5^ cells/well) were seeded in 24-well plates and cultured for 24 h. Next, heat-killed *C. acnes* RT 5 diluted in DMEM (1 × 10^7^ CFU/mL), chlorogenic acid (Arshine Food Additives Co., Ltd.; Changsha, China), and taurine (Qianjiang Yongan Pharmaceutic; Qianjiang, China) were incubated at appropriate concentrations (chlorogenic acid: 5, 10; taurine: 500, 1000 μg/mL) with the cells for 24 h. The incubated supernatants were collected and stored at −20 °C. The expression levels of IL8 and TIMP2 were evaluated via ELISA, using the Human IL8/CXCL8 DuoSet ELISA Kit (DY208) and Human TIMP2 DuoSet ELISA Kit (DY971, R&D systems, Minneapolis, MN, USA), as per the manufacturer’s protocol.

### 2.5. Wound-Healing Assay

HaCaT cells (3.5 × 10^4^ cells/well) were seeded in Culture-Insert 2 Well 24 (ibidi; Munich, Germany) and allowed to attach and grow for 24 h. After removing the insert, chlorogenic acid and taurine were added at appropriate concentrations (chlorogenic acid: 5, 10; taurine: 500, 1000 μg/mL) and incubated in a humidified environment with 5% CO_2_ and 95% air at 37 °C. The gap was photographed at 0 and 16 h with an EVOS™ FL Auto 2 imaging system. The gap area was analyzed using the ImageJ/Fiji^®^ (Version: 1.51j8) plugin called Wound_healing_size_tool (Version: 1.0, NIH, Bethesda, MD, USA) [25].

### 2.6. mRNA and Protein Expression Analysis

HaCaT cells (1 × 10^5^ cells/well) were seeded in 24-well plates and cultured for 24 h. Next, chlorogenic acid (Arshine Food Additives Co., Ltd., Shenzhen, China), and taurine (Qianjiang Yongan Pharmaceutical Co., Ltd, Qianjiang City, China) were added at appropriate concentrations and the cells were incubated for 24 h. To quantify the mRNA expression level, we conducted real-time PCR analysis. First, following the manufacturer’s instructions, total RNA was extracted using an RNA extraction kit (AccuPrep^®^ Universal RNA Extraction Kit, Bioneer; Daejeon, Republic of Korea). The purity of the extracted RNA (A260/A280) was confirmed using nanodrop. After RNA extraction, cDNA was synthesized by reverse transcription using the AccuPower^®^ RocketScript™ Cycle RT PreMix (Bioneer) on a PCR thermocycler (R&D systems), according to the manufacturer’s protocol. Real-time PCR analysis was performed using cDNA that was collected from control cells and cells treated with chlorogenic acid and taurine. The TaqMan probes used in this study were as follows: GAPDH assay id 4333764F; HAS3 assay id Hs00193436_m1, IL1A assay id Hs00899844_m1, IL1B assay id Hs01555410_m1, IL6 assay id Hs00985639. The TaqMan™ Universal Master Mix II, with UNG (Applied Biosystems; Waltham, MA, USA) was used. PCR reactions were performed on the ABI 7500 Real Time PCR system as per the manufacturer’s protocol. The resulting data were analyzed using ABI software (Version: 2.3).

To examine the protein expression levels, we conducted ELISA and western blot using incubated supernatants and cell lysates. The expression levels of IL1α, IL1β, and IL6 were evaluated via ELISA, using the Human IL-1 alpha/IL-1F1 DuoSet ELISA Kit (DY200, R&D Systems, Minneapolis, MN, USA), Human IL-1 beta/IL-1F2 DuoSet ELISA Kit (DY201, R&D systems, Minneapolis, MN, USA), and Human IL-6 DuoSet ELISA Kit (DY206, R&D systems, Minneapolis, MN, USA), as per the manufacturer’s protocol. The expression level of HAS3 was evaluated via western blotting. Cells were washed with ice cold PBS and lysed on ice in M-PER buffer (ThermoFisher Scientific, Waltham, MA, USA) supplemented with Complete™ protease inhibitor cocktail and phosphatase inhibitor (Roche, Basel, Switzerland). Next, 40 μg of protein was analyzed by western blotting with appropriate antibodies to evaluate protein expression; HAS3 (SC-365322, 1000:1 dilution, Santa Cruz, CA, USA), GAPDH (SC-47724, 2000:1 dilution, Santa Cruz, CA, USA). Western blot was analyzed by chemiluminescence detector (iBright FL1500, Invitrogen; Carlsbad, CA, USA). Western blot results were quantified by measuring band intensity using ImageJ analysis software (Version: 1.51j8; NIH, Bethesda, MD, USA) for three individual trials. The expression level was compared by comparing the intensity of HAS3 relative to GAPDH.

### 2.7. Immunocytochemistry

After 24 h of culture, cells were treated with appropriate concentrations of chlorogenic acid and taurine for 24 h. After an ice cold PBS wash, HaCaT cells were fixed with 4% paraformaldehyde at room temperature for 10 min. Cells were then permeabilized with PBS containing 0.1% Triton X-100 and subsequently blocked with PBS containing 5% FBS and 1% BSA. After incubation with the CLDN1 primary antibody (200:1 dilution, Abcam, ab211737, Cambridge, UK) at 4 °C for 12 h and the Alexa 594 nm conjugated secondary antibody (1000:1 dilution, A11012, ThermoFisher Scientific; Waltham, MA, USA) at room temperature for 1 h, the nucleus was stained with DAPI (2000:1 dilution, D1306, ThermoFisher Scientific; Waltham, MA, USA) in the dark for 10 min. High resolution fluorescence images were taken using the EVOS^TM^ FL Auto2 Imaging System (ThermoFisher Scientific, Waltham, MA, USA). Quantitative analysis of fluorescence intensity was performed using Celleste Image Analysis Software (Version: 4.1, ThermoFisher Scientific, Waltham, MA, USA). The entire part of the image was analyzed to extract red fluorescence intensities, and the number of pixels with fluorescence was counted to calculate both the intensity and area of fluorescence.

### 2.8. Assessment of Wound-Healing Efficacy on Human Skin

The study was conducted in accordance with the ethical standards of the Helsinki Declaration and was approved by the Institutional Review Board of Korea Institute of Dermatological Sciences (approval number KIDSIRB-2024-0121). To evaluate the wound-healing efficacy of chlorogenic acid and taurine, 31 healthy individuals (mean age, 48.13 ± 10.30) were recruited. Each participant provided written informed consent after being advised what to expect from the study. Subjects applied a specified amount of serum containing chlorogenic acid and taurine evenly to the facial area after washing twice daily for 4 weeks. All studies were carried out at consistent humidity and temperature levels (relative humidity: 50 ± 5%; temperature: 22  ±  2 °C). Measurements were performed after waiting for 30 min in a stable environment with steady humidity and temperature.

To evaluate the recovery efficacy of the wound area, we first induced skin irritation using Sodium Lauryl Sulfate (SLS). After placing the filter paper disc in a Fin Chamber (Smart Practice^Ⓡ^, Phoenix, AZ, USA) with a diameter of 12 mm, 60 µL of 2% SLS was dripped and affixed to the forearms. Attachment lasted for 24 h and after 24 h of patch removal, the Transepidermal Water Loss (TEWL) measurement was performed on the test material application area and the non-applied area using a Vapometer (Delfin Technologies Ltd., Kuopio, Finland).

Skin density was measured using a DUB Skin Scanner (Taberna pro medicum, Luneburg, Germany), by pressing a 3 cm area next to the left eye tail of all subjects with the same pressure.

### 2.9. Statistical Analysis

Experimental results are displayed as the average ± standard error of the mean (S.E.M.) from a minimum of three independent experiments. The results were analyzed using Excel (Microsoft, Redmond, WA, USA). The statistical significance was determined using the Student’s *t*-test. Values *p* < 0.05 were considered statistically significant.

## 3. Results

### 3.1. C. acnes Treated HaCaT Cells Released Different Wound-Healing Cytokines Based on Subtype

To evaluate the epidermal inflammatory cytokine properties impacted by *C. acnes*, heat-killed *C. acnes* RT1, RT4, RT5, and RT6 were added at a concentration of 1 × 10^7^ CFU/mL to HaCaT cells. Using the dot blot inflammatory cytokine array to evaluate a total of 40 targets, 29 cytokines were expressed by treatment with *C. acnes* subtypes (Table 1). In particular, from eosinophils, monocyte related chemoattractants (i.e., EOTAXIN1,2, I-309, MCP1,2, RANTES) were expressed [26,27,28,29]. I-309, MCP1, and MCP2, which recruit monocytes, were modestly decreased by treatment of HaCaT cells with RT1, RT4, RT5, and RT6, whereas eosinophil recruitment molecules such as EOTAXIN1, 2 showed a slight increment after treatment with RT5. RANTES were also upregulated by RT1, RT4, RT5, and RT6, indicating that the very first step of the wound repair process mediated by eosinophils was triggered by *C. acnes* (Table 1).

The proinflammatory cytokines IL1β, IL6, and IL8 were significantly upregulated mainly by RT1 and RT4 (Table 1). Although the proinflammatory cytokine recruitment process is an essential step for non-wounded cells to proliferate, a previous study reported that expression of IL1β and IL8, but not IL6, was associated with skin pigmentation [30,31]. The expression of other growth factors that were released shortly after the onset of the inflammation response such as GM-CSF and ICAM1 were upregulated by RT1 and RT5 [32] (Table 1). However, TIMP2, which is important for suppressing matrix metalloproteinase (MMP) expression during wound closure, was downregulated by RT1, RT4, RT5, and RT6 (Table 1). This result indicated that the *C. acnes*-triggered wound-healing process could induce an inflammatory response during cell proliferation, but also risk causing skin pigmentation and insufficient wound recovery [33,34,35].

**Figure 1:**
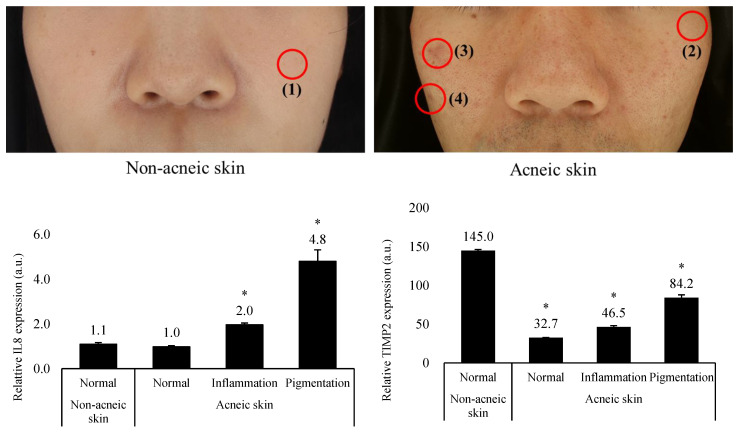
Comparative analysis of IL8 and TIMP2 expression in normal skin, acne-affected, inflamed, and pigmented sites. IL8 and TIMP2 protein expression were quantified from (1) non-acneic skin, (2) normal skin, (3) sites of inflammation, and (4) sites of pigmentation in acneic skin by ELISA. * *p* < 0.05 versus control group; Student’s *t*-test. Data are expressed as the mean ± SEM.

To evaluate the *C. acnes*-induced wound-healing process in the skin, we analyzed the expression of IL6, IL8, and TIMP2 in different sites of the cheek (Figure 1). Because IL6 was not detected in skin swabs due to the low detection limit, only the relative expression levels of IL8 and TIMP2 were measured in acneic skin compared to healthy (non-acneic) controls. Acneic individuals showed similar levels of IL8 in normal skin sites compared to non-acneic skin. However, a two-fold upregulation of IL8 expression was detected at sites of inflammation where acneic pimples were present. Moreover, 4.8-fold upregulated expression of IL8 was detected in pigmented spots where acneic pimples were removed and the repair processing was ongoing. In contrast, TIMP2 was highly expressed in non-acneic healthy skin, whereas acneic skin showed low levels of TIMP2 expression. Interestingly compared to normal sites in acneic skin, TIMP2 levels were upregulated by 1.4 fold at sites of inflammation and 2.6 fold at pigmented sites. The low level of TIMP2 in acneic skin indicated a low capacity for wound recovery that may lead to pigmentation and scar formation. However, at pigmented sites where wound recovery was ongoing, TIMP2 levels were upregulated, but not at similar levels as that in non-acneic skin.

Given these results, we hypothesized that IL8 and TIMP2 expression levels were key for the proper wound-healing process without the formation of scars or any pigmentation.

### 3.2. Chlorogenic Acid and Taurine Synergistically Regulate IL8 and TIMP2 Expression

**Figure 2:**
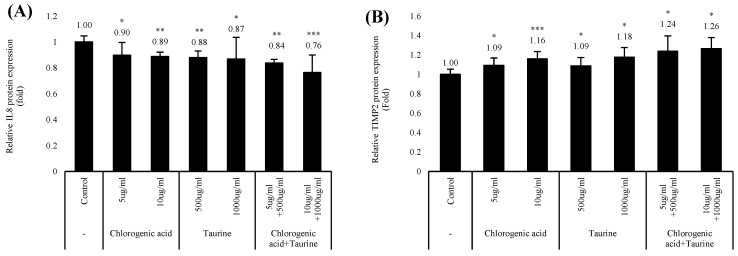
IL8 and TIMP2 regulatory effects of chlorogenic acid and taurine in cultured HaCaT cells. The synergistic effect of chlorogenic acid and taurine on (**A**) IL8 inhibition, and (**B**) TIMP2 activation after 24 h of co-treatment with heat-killed *C. acnes*. * *p* < 0.05, ** *p* < 0.01, *** *p* < 0.001 relative to the control group; Student’s *t*-test. Data are expressed as mean ± SEM.

To evaluate the wound-healing potential of chlorogenic acid and taurine, we analyzed the expression level of IL8 and TIMP2 in HaCaT cells treated with heat-killed *C. acnes*. IL8 expression was reduced to 90% and 89% with 5 μg/mL and 10 μg/mL chlorogenic acid treatment, respectively. Taurine reduced IL8 expression to 88% and 87% after treatment with concentrations of 500 μg/mL and 1000 μg/mL, respectively. The synergistic effect of IL8 inhibition was at 84% and 76% after treatment with a combination of 5 μg/mL and 500 μg/mL, and 10 μg/mL and 1000 μg/mL chlorogenic acid and taurine, respectively (Figure 2A).

TIMP2 levels were found to be elevated at 9% and 16% after treatment with 5 μg/mL, and 10 μg/mL chlorogenic acid, respectively. TIMP2 expression was upregulated by 9% and 18% after treatment with 500 μg/mL and 1000 μg/mL of taurine, respectively. Further, the synergistic effect on TIMP2 upregulation was evaluated after treatment with a combination of chlorogenic acid and taurine, and an increase in expression levels at 24% and 26% with 5 μg/mL and 500 μg/mL, and 10 μg/mL and 1000 μg/mL co-treatment was observed (Figure 2B).

### 3.3. Chlorogenic Acid and Taurine Accelerated the Wound-Healing Process and Enhanced Tight Junction Integrity

To evaluate the wound closure effect of chlorogenic acid and taurine, we performed a wound-healing assay and immunostaining for tight junction protein expression. Chlorogenic acid treated cells showed a wound-closure area of 133% and 140% when used at a concentration of 5 μg/mL and 10 μg/mL, respectively, compared to non-treated cells. The results for taurine treatment showed that a wound closure of 167% and 132% was observed after treatment with a concentration of 500 μg/mL and 1000 μg/mL, respectively.

**Figure 3:**
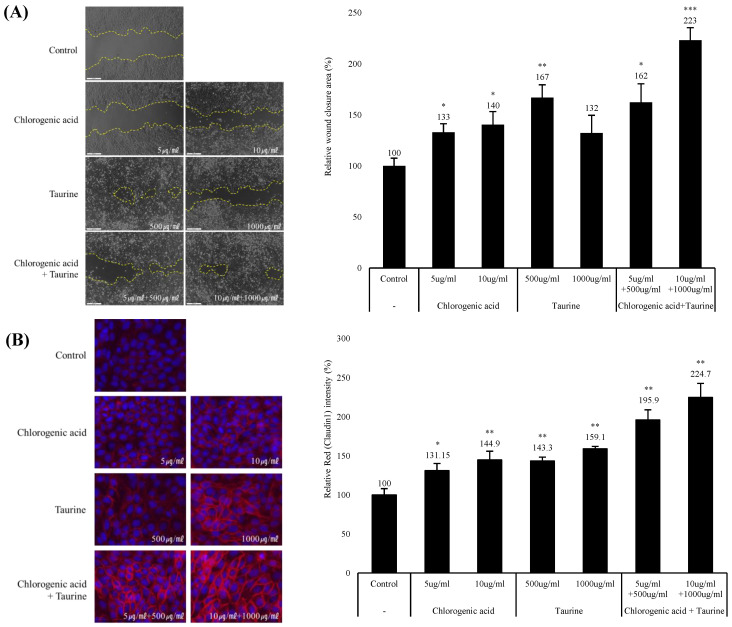
The synergistic effects of chlorogenic acid and taurine in wound closure and tight junction reinforcement in cultured HaCaT cells. (**A**) Synergistic wound-healing effect after 24 h treatment with chlorogenic acid and taurine treatment. (**B**) Upregulated claudin 1 expression after 24 h treatment was analyzed by immunostaining and fluorescence quantification. * *p* < 0.05, ** *p* < 0.01, *** *p* < 0.001 relative to the control group; Student’s *t*-test. Data are expressed as mean ± SEM.

In addition, co-treatment results showed a wound closure area of 162% and 223% after the administration of 5 μg/mL and 500 μg/mL, and 10 μg/mL and 1000 μg/mL of chlorogenic acid and taurine, respectively (Figure 3A).

Chlorogenic acid and taurine treatment caused increased expression of claudin 1, which may enhance cell-cell interactions after wound closure. Treatment with 5 μg/mL and 10 μg/mL chlorogenic acid resulted in 131% and 145% upregulation in claudin 1 expression. Additionally, treatment with 500 μg/mL and 1000 μg/mL taurine resulted in 143% and 159% upregulation in claudin 1 expression, respectively. The synergistic effect of the increased expression of the tight junction protein claudin 1 was analyzed after treatment with a combination of 5 μg/mL and 500 μg/mL, and 10 μg/mL and 1000 μg/mL chlorogenic acid and taurine, and an increase of 196% and 225% was observed, respectively (Figure 3B).

### 3.4. Chlorogenic Acid and Taurine Downregulated Inflammation Markers and Upregulated Hydration Marker Expression

Regulation of acute inflammatory cytokines and skin hydration levels are closely related to faster wound healing, scar, pigmentation, and epithelialization. Aquaporins regulate keratinocyte differentiation and proliferation through the activation of lipid metabolism and by accelerating glycerol transport [36]. Moreover, as a natural moisture retaining factor in the skin, hyaluronic acid synthesis is essential for epidermal structural stability during wound closure [37,38].

**Figure 4:**
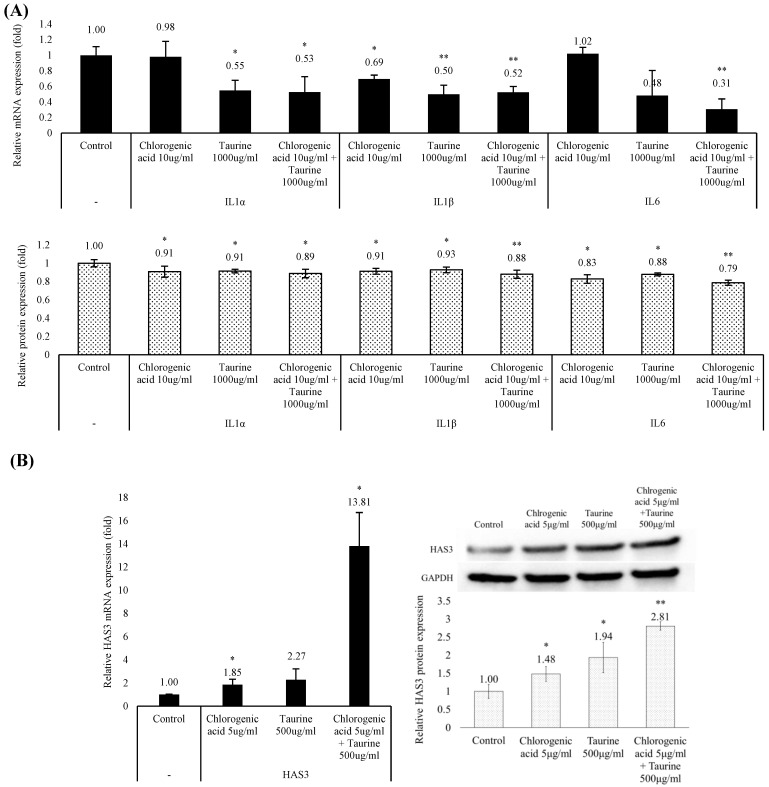
Regulatory effects of chlorogenic acid and taurine on inflammatory cytokines and hydration markers. Treatment with chlorogenic acid and taurine for 24 h (**A**) decreased the mRNA and protein expression level of *IL1α*, *IL1β*, and *IL6*, and (**B**) increased mRNA and protein expression of *HAS3* in cultured HaCaT cells. * *p* < 0.05, ** *p* < 0.01 relative to the control group; Student’s *t*-test. Data are expressed as mean ± SEM.

We identified several inflammatory cytokines that were downregulated by treatment with chlorogenic acid and taurine. In particular, *IL1α*, *IL1β*, and *IL6*, which performed a critical function during the initial phase of wound healing, were significantly reduced to 53%, 52%, and 31%, respectively, relative to control treatments (Figure 4A). Further, the mRNA and protein levels of hyaluronic acid synthase 3 (*HAS3*), which play a key role during the later phase of wound healing and matrix formation, were significantly increased to 1381% and 281%, respectively, relative to controls (Figure 4B). These results indicated that chlorogenic acid and taurine may play a significant role in the later stages of wound healing, specifically in the remodeling phase.

### 3.5. Chlorogenic Acid and Taurine Helped in the Recovery of Wounded Skin and Enhanced Skin Density

**Figure 5:**
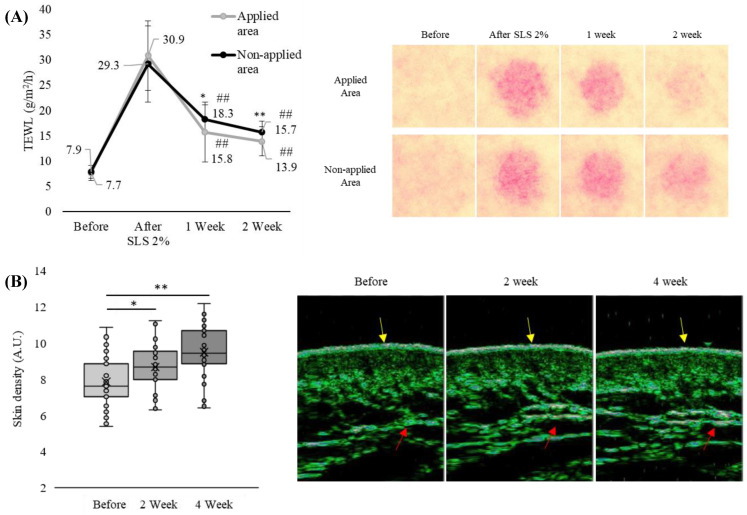
Attenuation of irritated epidermis and enhancement of dermal density by treatment of chlorogenic acid and taurine. (**A**) The restorative effect of chlorogenic acid and taurine on an SLS-induced skin wound. ## *p* < 0.01 relative to treatment with SLS. * *p* < 0.05, ** *p* < 0.01 comparing the SLS applied and non-applied group. (**B**) Increment in epidermal and dermal density by long-term treatment of chlorogenic acid and taurine (yellow arrows: epidermis, red arrows: dermis).* *p* < 0.05, ** *p* < 0.01 versus before group; Student’s *t*-test. Data are expressed as mean ± SEM.

To elucidate the wound-healing effect of chlorogenic acid and taurine, the forearm skin that was administered the skin irritant SLS (2%) was treated with a combination of 0.1% taurine and 0.001% chlorogenic acid. TEWL values were significantly reduced after 1 and 2 weeks of application of both chlorogenic acid and taurine compared to the untreated area. Application of the chlorogenic acid and taurine mixture resulted in a significant improvement in TEWL by 49% (untreated: 37%) after 1 week and 55% (untreated: 46%) after 2 weeks. Significant differences in TEWL were observed between the treated and untreated areas at both the 1-week and 2-week measurement time points (Figure 5A). Moreover, long-term application of up to 4 weeks showed a trend for increasing skin density as measured by skin ultrasound. The increase in skin density at 2 weeks (10%) and 4 weeks (21%) was statistically significant compared to the pre-application values. Measurements of the same area over 4 weeks showed an increase in epidermal density, which was particularly evident by the increase in the white area (yellow arrow) of the epidermal layer, and an increase in cell density (red arrow) of the dermis (Figure 5B).

## 4. Discussion

Acne scars are one of the most common forms of scars we encounter in daily life. The process of popping a pimple and its healing can be considered as an example of the wound-healing process. If acne scars are not treated properly, hyperpigmentation or facial scarring may occur [30]. In this study, we investigated the wound-healing effects of chlorogenic acid and taurine in vitro and in vivo, focusing on the modulation of inflammation factors.

We identified IL8 and TIMP2 as key factors for wound healing by analyzing the cytokine profile of *C. acnes* treated HaCaT cells in vitro (Table 1) and cotton swab samples of acne pimple scars site in vivo (Figure 1). At the sites of inflammation and pigmentation, IL8 expression levels were significantly increased compared to normal sites in acneic skin. Further, acneic skin showed relatively lower levels of TIMP2 expression, indicating a lower skin remodeling capability. TIMP2 expression gradually increased as wound healing progressed compared to that at normal skin sites. IL8 levels exhibited a continuous increase following acne wound formation, reaching its peak expression during the pigmentation phase, suggesting a correlation between inflammatory hyperpigmentation and elevated IL8 expression [30]. In acneic wound sites, TIMP2 expression was also increased, indicating an association with the wound-healing process; however, its levels remained lower compared to normal skin. This result suggests that even with an active wound-healing process, acne-prone skin may form incomplete skin-matrix structures compared to normal skin, leading to remaining scars (Figure 1). Although MCP1 expression was similarly high as TIMP2 and IL8, it did not exhibit an increasing trend in expression upon *C. acnes* stimulation. This could be attributed to *C. acnes* being a predominant skin commensal bacterium, because monocytes are thought to be more strongly activated by pathogens such as *Staphylococcus* according to the literature. Therefore, further investigation is warranted to understand the role of MCP1 in the skin [39].

Based on the known roles of IL8 and TIMP2 regulation in wound healing, we tested several natural compounds using the HaCaT cell model in vitro. Screening of several natural compounds showed that chlorogenic acid and taurine were effective modulators of both IL8 and TIMP2 mRNA and protein expression levels. Under heat-killed *C. acnes* treatment conditions, chlorogenic acid and taurine synergistically downregulated IL8 and upregulated TIMP2 protein expressions to normal levels (Figure 2A,B). However, among the compounds tested, only chlorogenic acid and taurine exhibited synergistic effects.

After the identification of the synergistic potential of chlorogenic acid and taurine in wound healing, we assessed their impact on factors directly implicated in the wound-healing process. Notably, the wound-healing assay conducted using HaCaT cells showed that chlorogenic acid and taurine effectively promoted cell proliferation, a crucial process in wound closure (Figure 3A). In wound healing, tight junctions between adjacent cells are crucial for robust wound closure. Therefore, the enhanced expression of cell-cell junction proteins induced by chlorogenic acid and taurine indicates their potential to promote wound repair (Figure 3B).

Previously, we showed that chlorogenic acid and taurine play a crucial role in wound healing. To identify the specific steps in the wound-healing process where these two substances are essential, we investigated the expression of key inflammatory factors and moisturizing-related factors (HAS), which is critical for tissue remodeling. Although a comprehensive assessment of all factors is still pending, the observed downregulation of several inflammatory factors and the increased expression of HAS3, major hyaluronan producers in the epidermis, suggests a potential role for chlorogenic acid and taurine in mitigating early stage inflammation and promoting wound closure at the wound site (Figure 4A,B) [40].

Although our initial observations focused on acne scars, to quantitatively assess wound healing, we compared the recovery of SLS-induced skin damage followed by natural healing and treatment with chlorogenic acid and taurine. In the earlier stage (after 1 week) of wound healing, skin treated with chlorogenic acid and taurine exhibited a significantly faster reduction in erythema and decreased transepidermal water loss (TEWL) compared to non-applied skin area. By 2 weeks, this difference became visually apparent, with treated wounds showing markedly reduced erythema and improved healing as assessed by both TEWL and visual measurements (Figure 5A). To assess the impact of chlorogenic acid and taurine on skin density, we conducted longitudinal ultrasound skin analysis over a 4-week treatment period tracking epidermal and dermal density changes within the same skin area. Results revealed a significant increase in both epidermal and dermal density at 4 weeks compared to baseline (0 weeks) and 2 weeks, indicating a progressive enhancement of skin density over time (Figure 5B).

We focused on finding a substance that could simultaneously regulate IL8 and TIMP2. We believed that inhibiting IL8 to terminate the inflammation step during the wound-healing stage and increasing TIMP2 to promote the proliferation or remodeling step is crucial in the wound-healing process. In particular, a prolonged inflammation step can exacerbate PIH through subsequent melanin formation. Additionally, insufficient expression of matrix formation factors can lead to scar formation or uneven skin recovery. In this context, we have indirectly confirmed that a substance capable of simultaneously inhibiting IL8 and increasing TIMP2 can be a key factor in regulating the wound-healing process during skin recovery.

## 5. Conclusions

**Figure 6:**
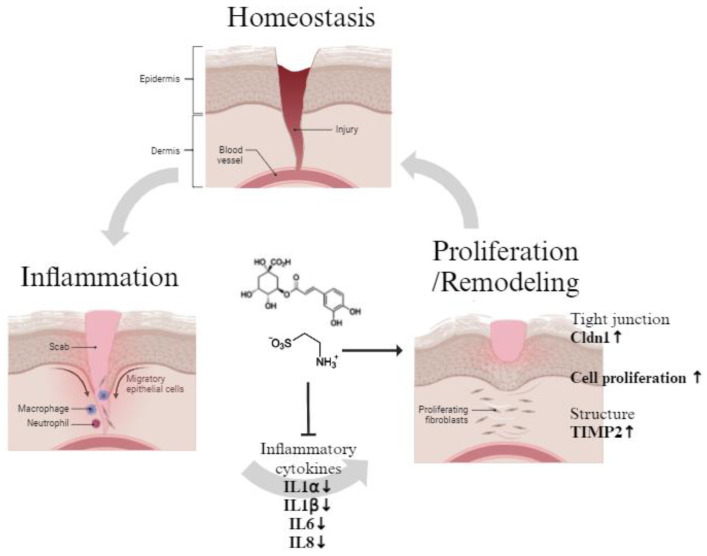
Summarized effects of chlorogenic acid and taurine in wound-healing effects (↑: activation, ↓: suppression).

We investigated the wound-healing potential of two promising natural compounds. Inspired by acne scar treatment, we initially screened the in vitro efficacy of various natural compounds and subsequently selected chlorogenic acid and taurine due to their synergistic effects. Our findings suggest that combinatorial treatment with these two substances effectively promotes skin wound healing by suppressing the inflammatory reaction, an early step in the wound-healing process, and activating proliferation and remodeling [41] (Figure 6). Although further research is needed to elucidate their precise functions, this study demonstrates the potential of chlorogenic acid and taurine as wound-healing activators.

## Figures and Tables

**Table 1:** Inflammatory cytokine/chemokine profiles induced by *C. acnes* subtypes.

	Control	*C. acnes*			
		RT1	RT4	RT5	RT6
EOTAXIN	4269	2807	2577	6098 *	3016
EOSTAXIN-2	4822	3549	2978	5599 *	3769
GM-CSF	1374	3027 *	1024	3119 *	1402
ICAM-1	2204	3103 *	1622	3481 *	1728
IFN-γ	1977	2050	1379	2469	1512
I-309	1035	749 *	627*	1144	625
IL1β	926	1011	1452	1866 *	1435
IL2	568	551	713	735	672
IL3	1768	2269	2732	2510	2465
IL4	930	830	938	938	858
IL6	795	18,243 **	971	15,600 **	1189
IL6sR	2952	3540	2142	4445	2164
IL7	777	551	538	612	635
IL8	20,736	27,118 **	17,180 *	24,431 **	19,036
IL10	3451	1877	1386	3660	1871
IL11	1422	1379	1161	1517	1265
IL12p40	1547	1884	1441	2756 *	1395
IL12p70	1717	1804	1444	1889	1102
IL13	204	385	469	430	477
IL15	1699	1629	1519	3449 *	1768
IL16	1663	1832	1640	2085	1919
IL17	1398	1350	1280	1356	1427
IP10	11,672	7293	5679	10,349	7650
MCP1	28,997	27,476	21,413 *	24,417 *	26,040
MCP2	1689	1064 *	869 *	1478	1150 *
RANTES	1755	3204 *	2661 *	3908 *	4113 *
TNF-α	2969	1731	1316	2285	1861
TNF-β	12,637	6323 *	5839 *	9101 *	7487 *
TIMP2	23,746	18,691 **	17,167 **	19,875 *	19,082 *

Note: HaCaT-releasing cytokine and chemokine profiles were analyzed by using arrays after 24 h of incubation with heat-killed *C. acnes* subtypes (RT1, RT4, RT5, RT6). Relative dot blot intensities were displayed by ImageJ (v1.51j8) analysis. * *p* < 0.05, ** *p* < 0.01 versus “Control” group; Student’s *t*-test.

## Data Availability

The datasets used and/or analyzed during the current study are available from the corresponding author upon reasonable request. Some data may not be available because of the policy of the company and ethical restrictions.
